# Higher Short-Chain Fermentable Carbohydrates Are Associated with Lower Body Fat and Higher Insulin Sensitivity in People with Prediabetes

**DOI:** 10.3390/nu15245070

**Published:** 2023-12-11

**Authors:** Natural H. S. Chu, Jie He, Kathy H. T. Leung, Ronald C. W. Ma, Jimmy Y. S. Lee, Jane Varney, Juliana C. N. Chan, Jane G. Muir, Elaine Chow

**Affiliations:** 1 Department of Medicine & Therapeutics, The Chinese University of Hong Kong, Prince of Wales Hospital, Hong Kong SAR, China; naturalchu@cuhk.edu.hk (N.H.S.C.);; 2 Li Ka Shing Institute of Health Sciences, The Chinese University of Hong Kong, Prince of Wales Hospital, Hong Kong SAR, China; 3 Hong Kong Institute of Diabetes and Obesity, The Chinese University of Hong Kong, Prince of Wales Hospital, Hong Kong SAR, China; 4 Department of Gastroenterology, Monash University and Alfred Hospital, Melbourne, VIC 3004, Australia

**Keywords:** diabetes, FODMAPs, impaired glucose tolerance (IGT), obesity, body fat, insulin sensitivity

## Abstract

The quality of carbohydrates has metabolic consequences in people with prediabetes. However, the causality of short-chain fermentable carbohydrate intakes and metabolic parameters has not been explored in the prediabetic or diabetic population. We investigated associations between different types of carbohydrates, including fermentable oligosaccharides, disaccharides, monosaccharides, polyols (FODMAPs), and polysaccharides (dietary fibre), and body composition and glucose/insulin responses in subjects with prediabetes. In this prospective cross-sectional study, 177 subjects with impaired glucose tolerance (IGT) (mean age: 60 (54–62) years, 41% men) underwent an assessment of body composition and completed six-point oral glucose tolerance tests (OGTT), Homeostatic Model Assessment of Insulin Resistance (HOMA2-IR), insulin sensitivity, detailed 3-day food records, and physical activity questionnaire. Daily habitual FODMAP intake decreased progressively with increasing BMI, ranging from 7.9 (6.2–12.7) g/d in subjects with normal BMI and 6.6 (4.6–9.9) g/d in subjects with overweight to 5.8 (3.8–9.0) g/d in subjects with obesity (*p* = 0.038). After adjustment for age and gender, galactooligosaccharides (GOSs) were negatively correlated with body fat (Standardised Beta coefficient β = −0.156, *p* = 0.006) and positively associated with insulin sensitivity (β = 0.243, *p* = 0.001). This remained significant after adjustment for macronutrients, fibre, and physical activity (*p* = 0.035 and *p* = 0.010, respectively). In individuals with IGT, higher dietary GOS intake was associated with lower body fat and higher insulin sensitivity independent of macronutrients and fibre intake, calling for interventional studies to evaluate the effect of FODMAP intake in prediabetes.

## 1. Introduction

Prediabetes is a high-risk state, with 5–10% of affected people progressing to diabetes annually. The prevalence of prediabetes is increasing worldwide, with more than 470 million people estimated to have prediabetes by 2030 [1,2]. Dietary intervention effectively prevents diabetes, and changes in gut microbiota may be implicated [3,4]. Lifestyle, dietary, and genetic factors can influence fat accumulation. Pooled analyses of three population-based studies suggested that the quantity and quality of carbohydrate intake are important determinants of weight change [5]. While low-grade inflammation in visceral adipose tissue can increase insulin resistance [6,7], the intestinal microbiome can modulate lipid metabolism through multiple mechanisms, including changes in short-chain fatty acids (SCFAs) [8,9]. 

FODMAPs (fermentable oligosaccharides, disaccharides, monosaccharides, and polyols) are fermentable short-chain carbohydrates metabolised by the gut microbiota. FODMAPs are sometimes confused with dietary fibre and prebiotics because both promote gut health and have some overlapping elements. Of FODMAP components, only galactooligosaccharides (GOSs) and fructooligosaccharides (FOSs) are included in the current definition of dietary fibre [10]. However, different types of carbohydrates may alter different metabolic pathways and the gut microbiota [11,12,13]. Accumulating evidence suggests that not all types of dietary fibre reduce insulin overload and T2D risk [14]. We hypothesise that FODMAP components have beneficial effects on body composition and insulin resistance, possibly via alterations in the gut microbiota. 

A low FODMAP diet reduces the symptoms of irritable bowel syndrome by 50–80% [15,16], but is accompanied by an increased abundance of *Bacteroides* and a reduced abundance of metabolically beneficial bacteria such as *Bifidobacterium* and *Akkermansia muciniphila* [17,18]. Through fermentation, these microbiota generate active metabolites which can alter the balance of deconjugated secondary bile acids [19], SCFAs [20], lipopolysaccharides (LPSs) [8], and incretin secretion [21,22], which are implicated in glucose and lipid metabolism. Supplementation with GOSs as prebiotics has been shown to alter body composition, gut hormones, and gut microbiota in animal studies [23,24]. FOSs added to a high-fat diet reduced body fat mass and adiposity in rats [25]. In another rodent study, GOS increased the incretin hormones glucagon-like peptide-1 (GLP-1) and peptide YY and the abundance of health-promoting *Bifidobacterium* [26]. In a double-blind, parallel randomised clinical trial (RCT) involving 44 subjects with overweight/obesity and prediabetes, 15 g of GOS daily, when added to a regular diet, increased *Bifidobacterium* by five times but did not alter peripheral insulin sensitivity, SCFAs, LPSs, or inflammatory markers [27]. However, compared with prebiotic supplementation, FODMAPs from whole foods might exert additional effects on whole gut transit time and motility [28] and interact with other gut components, resulting in different effects. However, human studies on FODMAP consumption and associations with body composition and metabolic indices in prediabetes are lacking.

In this study involving subjects with impaired glucose tolerance (IGT) participating in a diabetes prevention programme, we documented their habitual FODMAP intake including individual FODMAP components. We examined the cross-sectional associations between habitual FODMAP intake, body composition, and glucose/insulin response at baseline. We hypothesised that higher habitual FODMAP consumption is associated with lower body fat and more favourable insulin secretion and sensitivity profiles, independent of total macronutrient, fibre intake, and physical activity [29,30]. 

## 2. Materials and Methods

### 2.1. Participants

We utilised baseline cross-sectional data from participants with IGT who took part in a 12-month RCT which evaluated the effects of continuous glucose monitoring (CGM) as an adjunct to lifestyle modification for the prevention of glycaemic deterioration (NCT04588896). The study received ethical approval from the Joint Chinese University of Hong Kong-New Territories East Cluster clinical research ethics committee (CREC-2019.605). In this investigator-initiated, single-centre study conducted at the Prince of Wales Hospital (PWH), potential participants were identified from attendees at medical or general outpatient clinics or self-referral through advertisements. All participants provided written informed consent. Inclusion criteria included an age range of 18–65 years, a body mass index (BMI) range of 18–40 kg/m^2^, a non-pregnant or lactating state, and no history of diabetes and treatment with glucose-lowering or weight-reducing drugs. Subjects who participated in a weight-reducing programme within three months of screening were also excluded. After an overnight fast, all participants underwent a 75 g oral glucose tolerance test (OGTT). Glycaemic status and type 2 diabetes were defined according to the American Diabetes Association (ADA) criteria (2009): (1) normal glucose tolerance (NGT): fasting plasma glucose (FPG) < 5.6 mmol/L and 2-h PG < 7.8 mmol/L; (2) impaired fasting glycemia (IFG): 5.6 mmol/L ≤ FPG ≤ 6.9 mmol/L; (3) impaired glucose tolerance (IGT): 7.8 mmol/L ≤ 2-h PG < 11.1 mmol/L; (4) diabetes: FPG ≥ 7 mmol/L or 2-h PG ≥ 11.1 mmol/L. 

### 2.2. Anthropometrics, Body Composition, and Physical Activity

Anthropometric measures were taken with the subjects wearing light clothing and without shoes. Body weight and body composition (body fat percentage) were assessed by a bioelectric impedance analysis system (TBF-410 Body composition analyser, Tanita Corporation, Tokyo, Japan) [31,32]. Waist and hip circumferences (cm) were measured using a standard, retractable, non-metallic tape measure placed around the waist at the level of the umbilicus, across the largest part of the buttocks, and below the iliac crest. Height was measured with a stadiometer to the nearest 0.1 cm for the calculation of BMI. According to the World Health Organisation (WHO) Asian classification for obesity [33], the cut-off point for overweight was 23 kg/m^2^ and 27 kg/m^2^ for obesity. Therefore, we defined normal weight as 18–22.9 kg/m^2^, overweight as 23–26.9 kg/m^2^, and obesity as ≥27 kg/m^2^. Physical and activity levels were recorded using the International Physical Activity Questionnaire (IPAQ) (Chinese version) [34].

### 2.3. Biochemical Profiles

Blood samples were collected through a venous catheter from an antecubital vein into vacutainer tubes containing EDTA (ethylenediamine tetraacetic acid) at 0, 15, 30, 60, 90, and 120 min for the determination of plasma glucose. Plasma C peptide (CP) concentration was measured by radioimmunoassay (Novo Nordisk, Copenhagen, Denmark). The lowest detection limit was 0.1 nmol/L, with an intra-assay coefficient of variation (CV) of 3.4% and an inter-assay CV of 9.6% [35]. 

We computed the steady state of insulin resistance and dynamic indices of beta cell function using fasting CP and PG values. Insulin resistance (HOMA2-IR), insulin secretion (HOMA2-%B), and insulin sensitivity (HOMA2-%S) were calculated using The Homeostasis Model Assessment (HOMA2) Calculator v2.2.3. from http://www.dtu.ox.ac.uk, accessed on 23 June 2022 [36]. We analysed the HOMA2-IR score as a continuous value, with a high value indicating increased insulin resistance. We calculated PG and CP area under the curve (AUC) during OGTT. Early C-peptidogenic index was calculated by (30 min CP-fasting CP)/(30 min PG-fasting PG), and late C-peptidogenic index was calculated by (120 min CP-fasting CP)/(120 min PG-fasting PG).

### 2.4. Dietary Evaluation

Participants prospectively documented their habitual dietary intake using food records over three days at baseline prior to the allocation of study intervention. Each food record constituted one weekend and two weekdays to accurately capture variations in food intake between weekends and weekdays. Subjects were asked to provide details, including quantities of all consumed meals and beverages, according to standardised portion sizes published by the Centre for Food Safety Hong Kong [37]. Upon the return of the food records, the research dietitian carefully assessed the records to ensure all food items were correct with another research nutritionist’s confirmation. 

The food records were analysed for energy, macronutrient, and total dietary fibre content using a nutritional analysis programme (eSHA Food Analysis and Labelling Software: https://esha.com/, accessed on 1 February 2020 and FoodWorks 10—Xyris). The contents of individual FODMAPs, excess fructose (i.e., fructose in excess of glucose), lactose, fructans, GOSs, and polyols (sorbitol and mannitol) were calculated using the published Monash University FODMAP composition database (The Monash FODMAP calculator Melbourne, Australia) [38]. Regarding estimating the FODMAP content of commonly consumed food brands in Hong Kong, the first author arranged the collection and shipment of Hong Kong foods to Monash University for FODMAP analysis. Foods undergoing analysis included noodles (wheat and rice-based), rice, legumes, herbal tea, broth, vegetables, soft drinks, instant desserts, and a range of sauces. These data are available in the Monash FODMAP calculator and reported in publications [39,40].

Prospective 3-day food records have been used for dietary capture in observational and interventional studies of individuals with prediabetes and IGT [41,42]. This was shown to track with progression and regression from prediabetes. Alternative instruments such Food Frequency Questionnaires (FFQs), which are based on dietary recall, found weak associations between diet habits and glucose tolerance in 988 subjects [43]. In a meta-analysis of studies using FFQ in prediabetes, test–retest reliability ranged from 0.33 to 0.92 and relative validity ranged from 0.08 to 0.83 [44], highlighting the limitations of FFQs as a disease-specific instrument for prediabetes. Further, a 3-day food record has been shown to adequately rank an individual’s habitual information of FODMAPs [45] in a non-interventional setting. 

### 2.5. Statistical Analysis

#### 2.5.1. Descriptive Analyses

For comparisons, Student’s *t*-test, Mann–Whitney U test, chi-square (χ2), Fisher’s exact test, or analysis of variance (ANOVA) were used as appropriate. Post-hoc Tukey tests were performed for between-group comparisons if the overall results were significant. The non-parametric Kruskal–Wallis test was used to compare between-group differences in dietary intake of macronutrients and individual FODMAPs. If significance was detected, post hoc analysis of significant values was performed using the least significant difference test between the two subject groups. Values were reported as mean (95% confidence interval) for parametric data or median (interquartile range) for non-parametric data.

#### 2.5.2. Association Analyses

We used Spearman and Pearson correlation analysis to study relationships between total and individual FODMAP intake with body composition (BMI and total body fat) and between total and individual FODMAP items. Following checking of assumptions of normality, linearity, homoscedasticity, and absence of multicollinearity, we then constructed a multivariate linear model with body composition as the dependent variable and FODMAP items as the independent variable. We adjusted for age and sex as covariates in the base model. In model 1, we additionally included daily consumption of macronutrients and fibre intake as covariates, and in model 2, we additionally included physical activity. In model 3, we additionally adjusted for antihypertensive use as a covariate on top of model 2. We also examined the association of FODMAP intake with insulin response and insulin sensitivity adjusted for the aforementioned covariates. 

All data were analysed using the Statistical Package for Social Sciences version 26.0 (SPSS Inc., Chicago, IL, USA). The R-software version 4.3.2 package was used to calculate the correlation between FODMAPs and insulin indexes.

## 3. Results

### 3.1. Baseline Characteristics of the IGT Cohort

Between December 2020 and February 2022, we screened 502 subjects for eligibility using 75 g OGTT. Of these, 177 subjects were confirmed to have IGT, 250 had normal 2 h PG, and 64 had undiagnosed diabetes. All subjects with IGT completed a 3 day food record and other measurements. 

Table 1 summarises the characteristics of the study population. Amongst the 177 participants with IGT included in this analysis, 81% of subjects with IGT were either overweight or obese. A total of 59% were female with a median (IQR) age of 60 (54–62) years and BMI of 26.8 ± 3.9 kg/m^2^. A total of 42.9% were treated with antihypertensive drugs and 37.3% with statins.

### 3.2. Comparison of Baseline Characteristics by BMI Categories

Of all participants, 19% (n = 34), 40% (n = 70) and 41% (n = 72) were classified as normal, overweight, or obese, respectively. Total energy intake was similar between the BMI categories, as were carbohydrate, protein, and fat intake. There were no between-group differences in macronutrient intake except for a lower fibre intake amongst subjects with obesity (Table 2). Moreover, total physical activity levels differed amongst the three groups (*p* < 0.018) (Table 1). There were no differences in fasting, 1 h, or 2 h glucose between the BMI categories; however, % body fat and insulin sensitivity (HOM2%S) were significantly lower in the obese group, while early peptidogeneic index and 2 h C peptide were significantly higher in the obese group. 

### 3.3. Correlation between FODMAP Intake and Body Composition

The median of total FODMAPs in the IGT cohort was 6.6 (IQR: 4.5–10.3) g/day. The distribution of individual FODMAPs was calculated (Table 2). Amongst individual FODMAP items, fructans had the highest consumption, followed by lactose and excess fructose. Daily habitual FODMAP intake was lower in people with a higher BMI, ranging from 7.9 (6.2–12.7) g/d in subjects with normal BMI and 6.6 (4.6–9.9) g/d in subjects with overweight to 5.8 (3.8–9.0) g/d in subjects with obesity (*p* = 0.038 Kruskal–Wallis Test). 

Total FODMAPs were negatively correlated with total body fat (r = −0.295, *p* < 0.0001). For individual FODMAPs, GOS (0.26 vs 0.48 g/day, *p* = 0.001) and polyol intake were significantly lower (0.3 vs 1.2 g/day, *p* = 0.001) in subjects with obesity than subjects with normal BMI. There were no significant differences in excess fructose or lactose intake between the analysed BMI categories (Table 2). GOSs were negatively correlated with body fat. Other individual FODMAP contents, such as fructans, excess fructose, and lactose were also significantly negatively correlated with body fat (Table 3).

### 3.4. Correlation between FODMAP and Indices of Insulin Secretion and Resistance

There was no correlation between glucose response and FODMAP contents (Table 4). Among the individual FODMAPs, GOSs were negatively correlated with HOMA2-IR, HOMA2-beta, and early C-peptidogenic index, but positivity correlated with insulin sensitivity (HOMA2-S%). Fructans and mannitol were positively correlated with insulin sensitivity. Fructans and excess fructose were positively correlated with late C-peptidogenic index.

### 3.5. Multivariate Analysis of FODMAP Intake and Associations with Body Composition, Insulin Secretion, and Insulin Resistance

Next, we considered whether FODMAP contents predicted body composition independent of other covariates, including age, gender, total energy, macronutrients, and physical activity. As expected, we observed correlations between % body fat and total energy, carbohydrate, protein, fat, sugar, and fibre intakes (Supplementary Table S1). In our multivariate analysis, GOSs were associated with % body fat following adjustment for age and sex (β = −0.156, *p* = 0.006); however, these associations were attenuated following adjustment for total energy, carbohydrate, fat, protein, sugar, and fibre intakes as covariates (Table 5). The relationship was attenuated but remained significant (β = −0.131, *p* = 0.035) following further adjustment for physical activity. The association was attenuated after adjusting for antihypertensive drug use. 

We further considered associations between GOS intake and insulin sensitivity (HOMA2-S%). These remained significant following adjustment for macronutrients and fibre (β = 0.206, *p* = 0.006 and β = −0.174, *p* = 0.026) as well as physical activity (β = 0.211, *p* = 0.010 and β = −0.178, *p* = 0.032). In multivariate analysis, GOSs were significantly associated with insulin sensitivity (β = 0.243, *p* = 0.001) and postprandial 2 h CP (β = −0.202, *p* = 0.008) after adjustment of age and gender. The association was unchanged after adjustment for antihypertensive use (Table 5).

## 4. Discussion

In this study of Chinese individuals with IGT, we found an independent association of individual FODMAP consumption with lower body fat, lower postprandial 2 h CP, and higher insulin sensitivity after adjustment for macronutrients, fibre intake, and physical activity, with the strongest associations observed with GOSs.

We observed a low consumption of FODMAP in our study cohort (median 6.6 (4.5–10.3) g/day). Although not directly comparable, patients with IBS from the United Kingdom, Australia, the United States, Sweden, and New Zealand reported a daily intake of 16–31 g [46,47,48,49,50]. We and others have shown that South and East Asians generally have lower FODMAP consumption than their European counterparts in both the general population and those with IBS [51,52]. Despite these differences, no reports compare habitual FODMAP intake between Asian and European populations with prediabetes or diabetes. Currently, there are no international guidelines or recommendations for daily FODMAP intake [53]. 

Amongst Chinese subjects with IGT, subjects with obesity had the lowest intake of fibre content (subjects with obesity: 9 g/day; subjects with overweight: 12 g/day; and subjects with normal BMI: 16 g/day). Fibre has been associated with weight loss and insulin response independently of macronutrient and caloric intake in subjects with obesity [54,55]. Interestingly, apart from fibre and minerals [56], we also found robust associations between habitual consumption of individual FODMAPs and body fat and insulin sensitivity, independent of total calorie, macronutrient, or fibre intake and physical activity. These short-chain fermentable carbohydrates regulate adipocyte differentiation with reduced ectopic fat accumulation and improved lipid metabolism shown in an animal study [47]. Furthermore, FODMAPs are poorly digested or slowly absorbed [57,58] as demonstrated by new telemetric gas- and pH-sensing capsule technologies [59]. Enteroendocrine cells are present in the jejunum, and their greatest numbers are seen in the ileum and colon [60]. Fermentation of these short-chain carbohydrates stimulates the activity of SCFA-producing bacteria, such as *Roseburia*, *Akkermanisa*, and *Bifidobacterium*, which increase the production of SCFAs [28]. SCFAs are sensed by specific membrane receptors which may modulate immune responses to reduce inflammation factors, such as LPS, interleukin 6 (IL-6), and high-sensitivity C-reactive protein (hsCRP) [61], and stimulate endocrine receptors with increased gene expression of satiety-related peptides GLP-1, peptide YY, and proglucagon [62]. These changes in the metabolic and hormonal milieu can systemically regulate gluconeogenesis and glycogenolysis in the liver and lipolysis from adipose tissues [22,63,64]. In addition, these hormones work on the brain–gut axis to control food intake via increasing epigastric fullness and satiety [65,66].

In our study, GOSs were negatively correlated with BMI and insulin resistance, which might have contributed to the lower 2 h PG response associated with higher GOS intake. GOSs are a major prebiotic that reduces metabolic endotoxemia and improves human glucose tolerance [67]. GOSs can be a natural extract from indigestible carbohydrate materials or synthetically produced in various forms, presented as liquid (syrup), capsules, or natural foods. As a prebiotic supplementation, they were not shown to improve metabolic indices in a human study [27]. However, in healthy subjects, a 24 h high-FODMAP diet reduced lipopolysaccharide (LPS)-binding protein compared to the consumption of a low-FODMAP diet [68]. To this end, chronic subclinical inflammation and LPSs are known to increase insulin resistance [68]. Our study found a favourable association between high dietary short-chain fermentable carbohydrate intake, including GOSs, and weight loss and insulin metabolism. These differences might be due to the slower regional transit time of GOSs from natural foods [69,70] with increased contact time with other gut microbiota and possible interactions with other FODMAPs in contrast to the administration of GOS as a prebiotic supplement.

This is the first study investigating the metabolic response of habitual FODMAP intake in people with prediabetes. Low habitual FODMAP consumption may unfavourably influence gut microbiome diversity and predispose to metabolic diseases. In this study, we obtained detailed metabolic phenotyping with multiple-point OGTT and documentation of FODMAPs and macronutrients using a 3-day food diary and physical activity using a structured questionnaire. However, our study also had limitations. We did not assess body composition using dual-energy X-ray absorptiometry (DEXA), although bioimpedance analysis (BIA) is a validated measure of body composition, including body fat percentage, which closely correlates with that measured by DEXA and magnetic resonance imaging (MRI) [31,32]. Physical activity was only assessed using self-reported questionnaires, while the use of sealed pedometers and accelerometers may allow more accurate quantification. Only 1% of the study cohort were taking herbal, probiotic, or prebiotic supplements long-term (more than 3 months). We repeated our analyses after the exclusion of these individuals and found no effect on our results. Our sample size was relatively small. Nevertheless, our findings were robust and remained significant following adjustment for macronutrients, fibre, and physical activity, as fibre has been shown to improve insulin resistance and diabetes risk [14,71]. Since these individuals were recruited for a lifestyle modification programme, they might be more motivated, with higher health literacy, and their diets may not be generalisable. That being said, these data were collected at baseline before any intervention. Unmeasured variables, such as genetic factors, indigested proteins, and glycaemic index or load, might need to be clarified for our results. High FODMAP foods generally have a lower glycaemic index, with the total amount of carbohydrates in a food showing better correlations with diabetes risk than glycaemic index or load [58]. Furthermore, we recruited subjects from a population with high risk of chronic diseases; 42.9% of subjects took antihypertensive medication and these may affect the composition of the gut microbiota [72,73]. We additionally adjusted for antihypertensive drug use in our multivariate analysis, and this did not impact the association between GOSs and insulin sensitivity or secretion. Our results showed a weak-to-moderate correlation between GOSs and anthropometric variables and only explained a small proportion of variance for insulin sensitivity and secretion parameters. Finally, as association does not infer causation, our findings are hypothesis-generating; therefore, future interventional studies are needed to confirm the impact of FODMAP intake on metabolic outcomes in prediabetes.

## 5. Conclusions

Dietary FODMAP intake was low in Chinese subjects with IGT, especially those with obesity. Higher short-chain fermentable carbohydrate intake was associated with lower body fat and higher insulin sensitivity in the prediabetic cohort, independent of total energy, macronutrient, or fibre intake and physical activity. Our findings have potential implications for using FODMAPs as a specific dietary strategy to prevent or manage diabetes beyond calorie restriction and carbohydrate-restricted diets. Future studies are needed to better understand how dietary FODMAP components can influence metabolic responses and the gut microbiome.

## Figures and Tables

**Table 1:** Baseline demographics of subjects with IGT stratified by BMI categories.

Variable	Total Population (n = 177)	Normal (n = 34)	Overweight (n = 70)	Obesity (n = 73)	*p* Value	Missing Data (n)
Age, years	60 (54–62)	60 (55–63)	61 (58–63)	57 (51–61)	**0.008**	0
Male, n (%)	72 (41)	11 (32.4)	32 (45.7)	29 (40.7)	0.421	0
Weight, kg	70.6 ± 12.9	57.8 ± 7.7	67.3 ± 7.4	79.8 ± 12.5	**<0.0001**	0
Waist circumference, cm	93.5 ± 9.8	82.4 ± 5.4	91.4 ± 5.5	100.8 ± 8.8	**<0.0001**	0
Hip circumference, cm	99.8 ± 7.5	92.1 ± 4.1	97.1 ± 3.4	106.2 ± 6.5	**<0.0001**	0
BMI, kg/m^2^	26.8 ± 3.9	21.7 ± 1.4	25.4 ± 1.1	30.4 ± 2.9	**<0.0001**	0
Systolic blood pressure, mmHg	133 ± 16	125 ± 15	134 ± 15	136 ± 17	**0.048**	1
Diastolic blood pressure, mmHg	83 ± 10	79 ± 10	84 ± 10	84 ± 11	**0.027**	1
Body fat, %	31.8 ± 8.7	25.3 ± 6.1	29.4 ± 6.1	37.0 ± 8.8	**<0.0001**	2
Antihypertensive drug use, n (%)	76 (42.9)	12 (35.3)	25 (35.7)	39 (53.4)	0.061	0
Statin use, n (%)	66 (37.3)	9 (26.5)	30 (42.9)	27 (37.0)	0.268	0
Physical activities						
Vigorous, MET-min/week	0 (0–0)	0 (0–510)	0 (0–0)	0 (0–0)	0.560	2
Moderate, MET-min/week	0 (0–480)	240 (0–630)	120 (0–480)	0 (0–240)	**0.047**	3
Light, MET-min/week	693 (330–1386)	891 (322–1634)	693 (396–1386)	594 (289–1238)	0.172	11
Total physical activity MET-min/week	1166 (484–2243)	1569 (880–2891)	1181 (690–2233)	693 (297–2079)	**0.018**	1
Sedentary, min/day	300 (180–480)	240 (180–375)	300 (180–465)	360 (180–480)	0.331	17
Glycaemic indices						
Fasting plasma glucose, mmol/L	5.3 ± 0.5	5.3 ± 0.5	5.4 ± 0.5	5.4 ± 0.5	0.795	0
1 h plasma glucose, mmol/L	10.9 ± 1.6	11.0 ± 1.5	10.8 ± 1.7	11.0 ± 1.6	0.778	2
2 h plasma glucose, mmol/L	8.4 ± 1.4	8.3 ± 1.4	8.4 ± 1.5	8.5 ± 1.4	0.709	0
AUC-PG, mmol.L^−1^.min^−1^	18.5 ± 1.9	18.6 ± 1.6	18.4 ± 1.9	18.7 ± 2.0	0.638	2
Fasting plasma C-peptide, pmol/L	563 (434–742)	333 (270–458)	518 (431–651)	728 (579–827)	**<0.0001**	2
2 h plasma C-peptide, pmol/L	2955 (2295–3757)	2339 (1986–2900)	2774 (2274–3790)	3491 (2731–3914)	**<0.0001**	2
HOMA2-IR	1.27 (0.94–1.67)	0.74 (0.61–1.02)	1.16 (0.94–1.50)	1.63 (1.31–1.86)	**<0.0001**	2
HOMA2- β (%)	99.9 (77.0–125.5)	74.0 (55.2–87.8)	99.8 (73.9–117.6)	116.1 (97.4–142.9)	**<0.0001**	2
HOMA2-S (%)	78.4 (59.0–104.1)	135.2 (97.6–163.6)	86.2 (64.5–105.0)	61.3 (53.8–76.5)	**<0.0001**	2
Early C-peptidogenic index (pmol/mmol)	228 (163–333)	178 (135–205)	235 (180–353)	258 (185–377)	**0.001**	5
Late C-peptidogenic index (pmol/mmol)	718 (551–1030)	741 (466–976)	702 (539–1107)	723 (584–1019)	0.405	5

Mean ± SD, median (interquartile range). Non-parametric Kruskal–Wallis test or parametric one-way ANOVA were used for testing significance; Pearson chi-square for gender, antihypertensive drugs, and statin; significant values are in bold (*p* < 0.05). Abbreviations: BMI: body mass index, HOMA2-IR: Homeostatic Model Assessment for Insulin Resistance, HOMA2-B: Homeostatic Model Assessment for Beta cell function.

**Table 2:** Median consumption of macronutrients and FODMAPs by BMI categories.

	Total Population (n = 176)	Normal BMI > 18 and <23 kg/m^2^ (n = 34)	Overweight BMI 23–26.9 kg/m^2^ (n = 70)	Obesity BMI ≥ 27 kg/m^2^ (n = 72)	*p* Value
Macronutrients					
Energy, kcal/day	1885 (1553–2182)	1782 (1544–2112)	1966 (1563–2357)	1857 (1497–2122)	0.264
Carbohydrates, g/day	201 (165–248)	194 (157–233)	208 (158–256)	200 (167–244)	0.700
Protein, g/day	87 (72–102)	85 (69–101)	88 (80–103)	84 (67–102)	0.141
Fat, g/day	79 (61–96)	75 (59–96)	81 (62–100)	78 (59–94)	0.516
Sugar, g/day	41 (29–58)	43 (34–58)	42 (29–56)	40 (25–65)	0.664
Fibre, g/day	11 (8–15)	16 (13–19)	12 (9–15)	9 (6–13)	**<0.0001**
Dietary glucose, g/day	7.7 (3.8–11.2)	10.2 (6.6–15.6)	7.5 (4.0–11.7)	5.7 (2.6–9.5)	**0.001**
Dietary fructose, g/day	6.3 (3.3–9.2)	8.6 (5.6–11.2)	6.4 (3.2–8.8)	4.7 (2.3–8.7)	**0.001**
FODMAPs					
Total FODMAPs, g/day	6.6 (4.5–10.3)	7.9 (6.2–12.7)	6.6 (4.6–9.9)	5.8 (3.8–9.0)	**0.038**
Excess fructose #, g/day	0.9 (0.4–1.5)	1.0 (0.6–1.7)	0.9 (0.3–1.5)	0.8 (0.3–1.3)	0.094
Polyols *, g/day	0.6 (0.2–1.5)	1.2 (0.4–1.7)	0.8 (0.4–1.5)	0.3 (0.1–1.0)	**0.001**
Fructans, g/day	1.8 (1.4–2.6)	2.0 (1.4–3.0)	2.0 (1.6–2.7)	1.7 (1.2–2.3)	**0.015**
GOSs, g/day	0.4 (0.2–0.7)	0.48 (0.28–1.25)	0.46 (0.26–0.90)	0.26 (0.14–0.53)	**<0.0001**
Lactose, g/day	1.6 (0.3–4.4)	2.3 (0.5–5.9)	1.4 (0.1–4.0)	1.9 (0.2–4.3)	0.182

# Excess fructose is defined as fructose minus glucose. * Polyols are the sum of mannitol and sorbitol. Non-parametric Kruskal–Wallis test was used for testing significance and significant values are in bold (*p* < 0.05).

**Table 3:** Spearman correlation coefficients of individual and total FODMAPs and anthropometrics.

Correlation Coefficient	Dietary Glucose	Dietary Fructose	Excess Fructose	Lactose	Sorbitol	Mannitol	Fructans	GOSs	Total FODMAPs
Age, years	r = 0.108, *p* = 0.158	r = 0.106, *p* = 0.162	r = 0.034, *p* = 0.655	r = −0.062, *p* = 0.411	r = −0.038, *p* = 0.619	r = −0.013, *p* = 0.865	r = 0.041, *p* = 0.591	r = 0.039, *p* = 0.612	r = −0.022, *p* = 0.773
Sex	r = 0.010, *p* = 0.898	r = −0.005, *p* = 0.949	r = −0.075, *p* = 0.320	**r = −0.202, *p* = 0.007**	r = 0.001, *p* = 0.992	r = 0.113, *p* = 0.137	r = −0.124, *p* = 0.101	r = 0.036, *p* = 0.638	**r = −0.202, *p* = 0.007**
Weight, kg	**r = −0.190, *p* = 0.012**	**r = −0.167, *p* = 0.027**	r = −0.051, *p* = 0.505	r = 0.110, *p* = 0.147	r = −0.092, *p* = 0.223	**r = −0.228, *p* = 0.002**	r = −0.056, *p* = 0.459	**r = −0.174, *p* = 0.021**	r = 0.026, *p* = 0.732
Waist circumference, cm	**r = −0.243, *p* = 0.001**	**r = −0.232, *p* = 0.002**	r = −0.144, *p* = 0.056	r = −0.016, *p* = 0.829	**r = −0.172, *p* = 0.022**	**r = −0.204, *p* = 0.007**	**r = −0.160, *p* = 0.034**	**r = −0.245, *p* = 0.001**	r = −0.132, *p* = 0.080
Hip circumference, cm	**r = −0.220, *p* = 0.003**	**r = −0.218, *p* = 0.004**	r = −0.144, *p* = 0.056	r = −0.031, p=0.686	**r = −0.152, *p* = 0.044**	**r = −0.220, *p* = 0.003**	r = −0.121, *p* = 0.110	**r = −0.229, *p* = 0.002**	r = −0.145, *p* = 0.054
BMI, kg/m^2^	**r = −0.260, *p* < 0.0001**	**r = −0.252, *p* < 0.0001**	**r = −0.165, *p* = 0.029**	r = −0.020, p=0.796	**r = −0.183, *p* = 0.015**	**r = −0.271, *p* < 0.0001**	r = −0.147, *p* = 0.051	**r = −0.291, *p* < 0.0001**	r = −0.145, *p* = 0.054
Body fat, %	**r = −0.216 *p* = 0.005**	**r = −0.234, *p* = 0.002**	**r = −0.190, *p* = 0.013**	**r = −0.206, *p* = 0.007**	r = −0.090, *p* = 0.242	r = −0.099, *p* = 0.196	**r = −0.224, *p* = 0.003**	**r = −0.192, *p* = 0.012**	**r = −0.293, *p* < 0.0001**

Spearman correlation was used in this analysis, and significant values are in bold (*p* < 0.05).

**Table 4:** Spearman correlation coefficients of individual and total FODMAPs and glucose, insulin secretion, and sensitivity indices.

Correlation Coefficient	Dietary Glucose	Dietary Fructose	Excess Fructose	Lactose	Sorbitol	Mannitol	Fructans	GOSs	Total FODMAPs
Fasting glucose, mmol/L	r = −0.066, *p* = 0.387	r = −0.073, *p* = 0.333	r = −0.056, *p* = 0.464	r = 0.094, *p* = 0.217	r = 0.014, *p* = 0.854	r = 0.013, *p* = 0.867	r = −0.033, *p* = 0.668	r = 0.075, *p* = 0.320	r = 0.051, *p* = 0.500
1 h PG, mmol/L	r = −0.108, *p* = 0.157	r = −0.106, *p* = 0.164	r = −0.080, *p* = 0.297	r = 0.118, *p* = 0.122	r = 0.027, *p* = 0.724	r = −0.072, *p* = 0.347	r = −0.094, *p* = 0.217	r = −0.072, *p* = 0.344	r = 0.024, *p* = 0.755
2 h PG, mmol/L	r = −0.037, *p* = 0.629	r = −0.056, *p* = 0.456	r = −0.031, *p* = 0.682	r = −0.079, *p* = 0.299	r = 0.070, *p* = 0.356	r = −0.003, *p* = 0.969	r = 0.053, *p* = 0.488	r = −0.056, *p* = 0.457	r = −0.065, *p* = 0.389
Fasting C-peptide, pmol/L	**r = −0.162, *p* = 0.033**	r = −0.140, *p* = 0.066	r = 0.002, *p* = 0.984	r = 0.003, *p* = 0.966	r = −0.040, *p* = 0.603	r = −0.142, *p* = 0.061	r = −0.144, *p* = 0.058	**r = −0.288, *p* < 0.0001**	r = −0.044, *p* = 0.563
2 h plasma C-peptide, pmol/L	r = −0.086, *p* = 0.261	r = −0.035, *p* = 0.645	r = 0.080, *p* = 0.296	r = −0.060, *p* = 0.434	r = −0.006, *p* = 0.936	r = −0.124, *p* = 0.103	r = −0.129, *p* = 0.089	**r = −0.291, *p* < 0.0001**	r = −0.059, *p* = 0.437
HOMA2-IR	**r = −0.159, *p* = 0.036**	r = −0.134, *p* = 0.077	r = 0.010, *p* = 0.899	r = 0.009, *p* = 0.906	r = −0.030, *p* = 0.699	r = −0.137, *p* = 0.070	r = −0.145, *p* = 0.055	**r = −0.281, *p* < 0.0001**	r = −0.038, *p* = 0.622
HOMA2-beta	r = −0.092, *p* = 0.226	r = −0.074, *p* = 0.333	r = −0.017, *p* = 0.823	r = −0.068, *p* = 0.371	r = −0.109, *p* = 0.151	**r = −0.165, *p* = 0.029**	r = −0.115, *p* = 0.130	**r = −0.318, *p* < 0.0001**	r = −0.102, *p* = 0.179
HOMA2-S, %	r = 0.128, *p* = 0.092	r = 0.109, *p* = 0.153	r = 0.019, *p* = 0.804	r = 0.018, *p* = 0.811	r = 0.059, *p* = 0.440	**r = 0.166, *p* = 0.028**	**r = 0.156, *p* = 0.039**	**r = 0.301, *p* < 0.0001**	r = 0.068, *p* = 0.373
Early C-peptidogenic index (pmol/mmol)	r = 0.048, *p* = 0.529	r = 0.053, *p* = 0.489	r = 0.109, *p* = 0.155	r = −0.046, *p* = 0.549	r = 0.079, *p* = 0.304	r = −0.061, *p* = 0.426	r = −0.032, *p* = 0.679	**r = −0.193, *p* = 0.011**	r = −0.001, *p* = 0.988
Late C-peptidogenic index (pmol/mmol)	r = 0.010, *p* = 0.899	r = 0.082, *p* = 0.282	**r = 0.155, *p* = 0.042**	r = 0.038, *p* = 0.622	r = 0.002, *p* = 0.978	r = −0.021, *p* = 0.783	**r = 0.165, *p* = 0.030**	r = 0.061, *p* = 0.422	r = 0.043, *p* = 0.578

Spearman correlation was used in this analysis, and significant values are in bold (*p* < 0.05). Abbreviations: PG = plasma glucose; CP = C-peptide; HOMA = Homeostasis model assessment for insulin resistance (HOMA-IR) and beta cell function (HOMA-beta) derived from fasting PG and CP.

**Table 5:** Multivariate analysis of the association between GOS consumption and body fat and insulin secretion/resistance indices.

Dependent Variable	Standardised Beta Coefficient	95% CI	*p* Value	Adjusted R^2^
Body fat (%)				
Base model	−0.156	[−4.273 to −0.732]	**0.006**	0.467
Model 1	−0.106	[−3.559 to 0.167]	0.074	0.497
Model 2	−0.131	[−4.057 to −0.148]	**0.035**	0.535
Model 3	−0.116	[−3.797 to 0.087]	0.061	0.548
HOMA2-S%				
Base model	0.243	[6.227 to 24.416]	**0.001**	0.081
Model 1	0.206	[3.855 to 22.187]	**0.006**	0.152
Model 2	0.211	[3.311 to 23.676]	**0.010**	0.136
Model 3	0.212	[3.524 to 23.597]	**0.008**	0.161
Postprandial 2 h CP (pmol/L)				
Base model	−0.202	[−570.756 to −88.413]	**0.008**	0.036
Model 1	−0.174	[−533.255 to −34.202]	**0.026**	0.062
Model 2	−0.178	[−551.027 to −24.811]	**0.032**	0.106
Model 3	−0.178	[−550.093 to −28.674]	**0.030**	0.122

Body fat, HOMA-IR, insulin sensitivity, fasting CP, and postprandial 2 h CP were included as dependent variables, with GOSs as the independent variable. Base model: GOSs as independent variable adjusted for age and gender; Model 1 = base model + daily intake of total energy (carbohydrates, protein, fats, fibre, and sugar); Model 2 = Model 1 + physical activities (vigorous, moderate, light exercise, and sedentary). Model 3 = Model 2 + antihypertensive drugs. Multivariate analysis was used and significant values are in bold (*p* < 0.05).

## Data Availability

The data presented in this study are available on request from the corresponding author. The data are not publicly available due to restrictions on data privacy.

## Supplementary Materials

The following supporting information can be downloaded at: https://www.mdpi.com/article/10.3390/nu15245070/s1, Table S1: Spearmen correlation coefficients of macronutrients and anthropometrics, biochemical profiles and insulin secretion/resistance indices. Table S2: Pearson correlation coefficients of individual and total FODMAPs and anthropometrics. Table S3: Pearson correlation coefficients of individual and total FODMAPs and glucose, insulin secretion and sensitivity indices.

## Abbreviations

BIA	bioimpedance analysis
BMI	body mass index
CP	C-peptide
DEXA	dual-energy X-ray absorptiometry
FODMAP	fermentable oligosaccharides, disaccharides, monosaccharides, and polyols
FOS	Fructooligosaccharides
GLP1	glucagon-like peptide-1
GOS	galactooligosaccharides
HOMA2-IR	Homeostatic Model Assessment 2 for Insulin Resistance
HOMA2-B	Homeostatic Model Assessment 2 for beta cell function
hsCRP	high-sensitivity C-reactive protein
IFG	impaired fasting glycaemia
IGT	impaired glucose tolerance
LPS	lipopolysaccharide
NGT	normal glucose tolerance
OGTT	oral glucose tolerance test
PG	plasma glucose
PYY	peptide YY
SCFAs	short-chain fatty acids
T2D	type 2 diabetes mellitus
