# Supplementary material for: Higher Short-Chain Fermentable Carbohydrates Are Associated with Lower Body Fat and Higher Insulin Sensitivity in People with Prediabetes
Source: Nutrients. 2023 Dec 11;15(24):5070. doi: 10.3390/nu15245070 (PMC10745595; doi:10.3390/nu15245070)
Supplement: Supplementary file 1 [file nutrients-15-05070-s001.zip › nutrients-2694555-supplementary.pdf]

Supplementary Table S1. Spearmen correlation coefficients of macronutrients and anthropometrics, biochemical profiles and insulin secretion/resistance indices.

Correlation coefficient	Total calories (kcal/d)	Carbohydrates, g/day	Protein, g/day	Fat, g/day	Sugar, g/day	Fiber, g/day	Vigorous, MET-min/week	Moderate, MET-min/week	Light, MET-min/week	Total PA min/week	Sedentary, min/day
Age, years	r = -0.111, p=0.146	r = -0.057, p=0.456	r = -0.079, p=0.294	**r = -0.182, p=0.016**	r = -0.035, p=0.648	r =0.142, p=0.061	r = -0.002, p=0.978	**r =0.171, p=0.024**	r =0.081, p=0.299	r =0.113, p=0.136	**r = -0.389, p<0.0001**
Sex	**r = -0.407, p<0.0001**	**r = -0.295, p<0.0001**	**r = -0.425, p<0.0001**	**r = -0.305, p<0.0001**	r = -0.132, p=0.080	r =0.090, p=0.233	r =0.072, p=0.345	r = 0.005, p=0.950	r =0.057, p=0.466	r =0.017, p=0.821	r = -0.055, p=0.491
Weight, kg	**r =0.287, p<0.0001**	**r =0.263, p<0.0001**	**r =0.184, p=0.015**	**r =0.208, p=0.005**	r =0.083, p=0.272	**r = -0.298, p=0.001**	r = -0.024, p=0.752	**r = -0.153, p=0.043**	r = -0.050, p=0.519	r = -0.136, p=0.073	**r =0.203, p=0.010**
Waist circumference, cm	r =0.123, p=0.104	r =0.125, p=0.099	r =0.023, p=0.765	r =0.092, p=0.225	r = -0.074, p=0.328	**r = -0.334, p<0.0001**	r = -0.032, p=0.676	**r = -0.192, p=0.011**	r = -0.057, p=0.462	**r = -0.176, p=0.019**	r =0.134, p=0.092
Hip circumference, cm	r =0.065, p=0.394	r =0.130, p=0.085	r = -0.050, p=0.508	r =0.018, p=0.815	r =0.048, p=0.531	**r = -0.335, p<0.0001**	r = -0.059, p=0.441	**r = -0.177, p=0.020**	r = -0.070, p=0.368	**r = -0.185, p=0.014**	r =0.114, p=0.151
BMI, kg/m^2	r =0.033, p=0.668	r =0.069, p=0.366	r = -0.044, p=0.558	r = -0.011, p=0.881	r = -0.014, p=0.851	**r = -0.371, p<0.0001**	r = 0.006, p=0.940	r = -0.129, p=0.089	r = -0.090, p=0.251	**r = -0.162, p=0.032**	r =0.081, p=0.311
Body fat, %	**r = -0.339, p<0.0001**	**r = -0.223, p=0.003**	**r = -0.405, p<0.0001**	**r = -0.252, p=0.001**	**r = -0.182, p=0.017**	**r = -0.185, p=0.015**	r = -0.019, p=0.806	r = -0.091, p=0.240	r = -0.053, p=0.502	r = -0.134, p=0.079	r =0.010, p=0.906
Fating glucose, mmol/L	r =0.123, p=0.105	r =0.042, p=0.578	r =0.138, p=0.069	r =0.103, p=0.173	r = -0.038, p=0.613	r = -0.018, p=0.810	r = -0.025, p=0.739	r = -0.165, p=0.029	r = -0.083, p=0.286	r = -0.089, p=0.243	r = -0.097, p=0.223
1hr PG, mmol/L	r =0.032, p=0.673	r = -0.023, p=0.765	r =0.076, p=0.321	r =0.052, p=0.497	r = -0.062, p=0.416	r = -0.086, p=0.262	r = -0.058, p=0.450	r = -0.154, p=0.044	r = -0.130, p=0.097	r = -0.121, p=0.111	r =0.064, p=0.427
2hr PG, mmol/L	r =0.020, p=0.791	r =0.026, p=0.733	r = -0.026, p=0.731	r =0.000, p=0.999	r = -0.016, p=0.836	r = -0.049, p=0.520	r = -0.147, p=0.053	r = -0.135, p=0.076	r =0.046, p=0.559	r = -0.132, p=0.181	r =0.088, p=0.269

Fasting C-peptide, pmol/L	r =0.077, p=0.313	r =0.072, p=0.346	r =0.036, p=0.642	r =0.083, p=0.277	r = -0.124, p=0.102	**r = -0.307, p<0.0001**	r = -0.144, p=0.058	**r = -0.235, p=0.002**	r = -0.030, p=0.706	**r = -0.194, p=0.010**	**r =0.174, p=0.029**
2hr plasma C-peptide, pmol/L	r = -0.081, p=0.288	r = -0.061, p=0.426	r = -0.077, p=0.315	r = -0.077, p=0.310	**r = -0.193, p=0.011**	**r = -0.251, p=0.001**	**r = -0.261, p=0.001**	**r = -0.189, p=0.013**	r = -0.042, p=0.591	**r = -0.184, p=0.015**	r =0.098, p=0.219
HOMA2-IR	r =0.087, p=0.255	r =0.082, p=0.282	r =0.044, p=0.568	r =0.088, p=0.247	r = -0.119, p=0.117	**r = -0.301, p<0.0001**	**r = -0.152, p=0.045**	**r = -0.247, p=0.001**	r = -0.029, p=0.717	**r = -0.200, p=0.008**	**r =0.165, p=0.038**
HOMA2-beta	r =0.015, p=0.849	r =0.056, p=0.463	r = -0.045, p=0.556	r =0.008, p=0.917	r = -0.096, p=0.206	**r = -0.262, p<0.0001**	r = -0.057, p=0.453	r = -0.113, p=0.139	r = -0.020, p=0.799	r = -0.112, p=0.142	**r =0.217, p=0.006**
HOMA2-S %	r = -0.083, p=0.276	r = -0.101, p=0.186	r = -0.031, p=0.684	r = -0.067, p=0.383	r =0.130, p=0.088	**r =0.288, p<0.0001**	r =0.119, p=0.119	**r =0.232, p=0.002**	r =0.044, p=0.580	**r = 0.185, p=0.014**	**r = -0.173, p=0.030**
Early C-peptidogenic index (pmol/mmol)	r =0.022, p=0.774	r =0.095, p=0.216	r =0.006, p=0.942	r =0.001, p=0.991	r = -0.015, p=0.842	r = -0.125, p=0.103	r = -0.121, p=0.116	r = -0.051, p=0.506	r =0.041, p=0.605	r = -0.047, p=0.540	r =0.051, p=0.529
Late C-peptidogenic index (pmol/mmol)	r = -0.070, p=0.357	r = -0.017, p=0.828	r = -0.058, p=0.445	r = -0.104, p=0.172	r = -0.048, p=0.528	r = -0.065, p=0.397	r = -0.065, p=0.396	r = -0.127, p=0.097	r = -0.072, p=0.358	r = -0.107, p=0.163	r =0.033, p=0.679

Spearmen correlation was used in this analysis, and bolded was significant (p<0.05). Abbreviations: PG=plasma glucose; CP= C-peptide HOMA=Homeostasis model assessment for insulin resistance (HOMA-IR) and beta cell function (HOMA-beta) derived from fasting PG and CP.

Supplementary Table S2. Pearson correlation coefficients of individual and total FODMAPs and anthropometrics

Correlation coefficient	Dietary glucose	Dietary fructose	Excess Fructose	Lactose	Sorbitol	Mannitol	Fructans	GOS	Total FODMAPs
Age, years	r =0.111, p=0.142	r =0.104, p=0.168	r =0.030, p=0.694	r = -0.096, p=0.206	r = -0.036, p=0.635	r = 0.006, p=0.941	r = -0.012, p=0.876	r = 0.031, p=0.679	r = -0.071, p=0.349
Sex	r =0.014, p=0.858	r = -0.004, p=0.954	r = -0.049, p=0.515	r = -0.138, p=0.067	r =0.026, p=0.730	r =0.138, p=0.069	r = -0.078, p=0.301	r =0.042, p=0.576	r = -0.111, p=0.142
Weight, kg	**r = -0.224, p=0.003**	**r = -0.194, p=0.010**	r = -0.096, p=0.203	r =0.039, p=0.607	r = -0.066, p=0.387	**r = -0.209, p=0.005**	r = -0.060, p=0.428	r = -0.138, p=0.067	r =0.054, p=0.479
Waist circumference, cm	**r = -0.265, p<0.0001**	**r = -0.246, p=0.001**	**r = -0.176, p=0.020**	r = -0.053, p=0.482	r = -0.129, p=0.088	**r = -0.230, p=0.002**	**r = -0.168, p=0.026**	**r = -0.201, p=0.007**	**r = -0.188, p=0.013**
Hip circumference, cm	**r = -0.230, p=0.002**	**r = -0.217, p=0.004**	**r = -0.191, p=0.011**	r = -0.073, p=0.337	r = -0.111, p=0.141	**r = -0.211, p=0.005**	r = -0.106, p=0.163	**r = -0.156, p=0.038**	**r = -0.174, p=0.021**
BMI, kg/m^2	**r = -0.257, p=0.001**	**r = -0.242, p=0.001**	**r = -0.178, p=0.018**	r = -0.068, p=0.372	r = -0.053, p=0.486	**r = -0.240, p=0.001**	r = -0.108, p=0.155	**r = -0.198, p=0.008**	**r = -0.169, p=0.025**
Body fat, %	**r = -0.212 p=0.005**	**r = -0.226, p=0.003**	**r = -0.204, p=0.007**	**r = -0.157, p=0.040**	r = -0.035, p=0.646	r = -0.076, p=0.323	r = -0.143, p=0.061	**r = -0.162, p=0.033**	**r = -0.229, p=0.003**

Pearson correlation was used in this analysis, and bolded was significant (p<0.05)Abbreivations: BMI body mass index

Supplementary Table S3. Pearson correlation coefficients of individual and total FODMAPs and glucose, insulin secretion and sensitivity indices

Correlation coefficient	Dietary glucose	Dietary fructose	Excess Fructose	Lactose	Sorbitol	Mannitol	Fructans	GOS	Total FODMAPs
Fasting glucose, mmol/L	r = -0.022, p=0.775	r = -0.038, p=0.614	r = -0.060, p=0.426	r =0.018, p=0.812	r =0.031, p=0.684	r =0.041, p=0.586	r = -0.003, p=0.971	r =0.061, p=0.420	r =0.019, p=0.804
1hr PG, mmol/L	r = -0.027, p=0.723	r = -0.038, p=0.618	r = -0.010, p=0.900	r =0.126, p=0.099	r = -0.001, p=0.995	r = 0.036, p=0.637	r = -0.074, p=0.333	r = -0.045, p=0.557	r =0.066, p=0.384
2hr PG, mmol/L	r = -0.036, p=0.636	r = -0.058, p=0.445	r = 0.016, p=0.830	r = 0.023, p=0.763	r =0.047, p=0.534	r = 0.050, p=0.509	r =0.040, p=0.596	r = -0.026, p=0.736	r = 0.042, p=0.581
Fasting C-peptide, pmol/L	**r = -0.187, p=0.013**	**r = -0.164, p=0.031**	r =-0.056, p=0.461	r =-0.035, p=0.648	r = 0.001, p=0.985	**r = -0.169, p=0.026**	r = -0.070, p=0.361	**r = -0.197, p=0.009**	r = -0.098, p=0.200
2hr plasma C-peptide, pmol/L	r = -0.112, p=0.140	r = -0.075, p=0.324	r =0.043, p=0.571	r = -0.040, p=0.602	r = 0.032, p=0.670	r = -0.100, p=0.191	r = -0.048, p=0.527	**r = -0.203, p=0.007**	r = -0.067, p=0.379
HOMA2-IR	**r = -0.187, p=0.013**	**r = -0.165, p=0.029**	r = -0.059, p=0.441	r = -0.035, p=0.649	r = 0.005, p=0.948	**r = -0.164, p=0.030**	r = -0.071, p=0.350	**r = -0.192, p=0.011**	r = -0.097, p=0.203
HOMA2-beta	r = -0.037, p=0.628	r = -0.087, p=0.256	r = -0.055, p=0.469	r = -0.048, p=0.529	r = -0.040, p=0.599	**r = -0.197, p=0.009**	r = -0.070, p=0.357	**r = -0.228, p=0.002**	r = -0.120, p=0.114
HOMA2-S, %	r =0.138, p=0.070	r =0.148, p=0.052	r =0.102, p=0.180	r =0.005, p=0.946	r =0.014, p=0.850	**r =0.174, p=0.022**	r =0.069, p=0.365	**r =0.251, p=0.001**	r =0.092, p=0.229
Early C-peptidogenic index (pmol/mmol)	r =0.027, p=0.727	r =0.039, p=0.611	r =0.067, p=0.382	r = -0.022, p=0.772	r =0.081, p=0.292	r = -0.145, p=0.058	r = 0.092, p=0.232	r = -0.085, p=0.270	r = 0.010, p=0.897
Late C-peptidogenic index (pmol/mmol)	r =0.025, p=0.745	r =0.036, p=0.639	r =0.084, p=0.270	r =0.010, p=0.895	r =0.058, p=0.450	r = 0.101, p=0.186	r = -0.013, p=0.867	r =0.036, p=0.637	r = 0.040, p=0.598

Pearson correlation was used in this analysis, and bolded was significant (p<0.05). Abbreviations: PG=plasma glucose; CP= C-peptide HOMA=Homeostasis model assessment for insulin resistance (HOMA-IR) and beta cell function (HOMA-beta) derived from fasting PG and CP.
